# Measurement invariance of the strength of motivation for medical school: a multi-group confirmatory factor analysis

**DOI:** 10.1186/s12909-017-0958-4

**Published:** 2017-07-11

**Authors:** M. An, R. A. Kusurkar, L. Li, Y. Xiao, C. Zheng, J. Hu, M. Chen

**Affiliations:** 1 Linyi University School of Education, Linyi, China; 2 Shantou University Medical College, 22 Xinling Road, Shantou, China; 3 VUmc School of Medical Sciences, Amsterdam, Netherlands

**Keywords:** Motivation, Medical school, Students, Measurement invariance

## Abstract

**Background:**

The Strength of Motivation for Medical School-Revised (SMMS-R) questionnaire measures students’ motivation for studying medicine. It includes three subscales: ‘willingness to sacrifice’, ‘readiness to start’, and ‘persistence’. Measurement invariance is a prerequisite for group comparisons. The objectives of this study were to verify the factorial structure of the SMMS-R questionnaire and to investigate it’s measurement invariance.

**Methods:**

A total of 989 medical students were approached, 930 cases were kept for data analysis. Factorial structure of and measurement invariance of the SMMS-R were tested using single and multiple group confirmatory factor analyses with Mplus. Trational Cronbach’s α along with McDonald’s ω and glb were used to measure internal consistency for each subscale.

**Results:**

Internal consistency for subscales and the full instrument were within the acceptable range. A 3-factor structure of the Chinese version of the SMMS-R was supported. Full configural, metric and partial scalar invariance were obtained.

**Conclusions:**

The SMMS-R showed measurement invariance across gender and two independent samples. So it can be used for group and cross-cultural comparisons.

**Electronic supplementary material:**

The online version of this article (doi:10.1186/s12909-017-0958-4) contains supplementary material, which is available to authorized users.

## Background

Motivation has been an important predictor of learning, academic success and well-being of medical students [1–3] There is evidence for studying the strength of motivation along with the quality of motivation in medical education [1, 4–7]. Strength of motivation has been positively correlated with autonomous (self-determined) forms of motivation and negatively correlated with non-self-determined forms of motivation and amotivation, and negatively correlated with exhaustion from study [8]. Also strength of motivation has been found to be higher in medical students admitted through weighted lottery selection in comparison with those admitted through a qualitative selection procedure [4, 6].

The Strength of Motivation for Medical School–Revised (SMMS-R) questionnaire, developed by Nieuwhof et al. [9] and revised by Kusurkar et al. [8], is the only instrument assessing students’ motivation specifically for medical studies. The SMMS-R measures the quantity of motivation for medical school and comprises three factors, willingness to sacrifice, readiness to start, and persistence [8, 10]. Willingness to sacrifice measures the willingness of a medical student to sacrifice his personal and social life in order to meet the time and effort demands of the medical study. Readiness to start measures the readiness and resolve to enter medical study. Persistence measures the will to continue medical study even in the face of difficult circumstances. The SMMS-R consists of 15 items and uses a 5-point Likert scale ranging from Strongly Disagree to Strongly Agree. Evidence has been reported for the reliability and validity of the scores of the SMMS-R subscales and the full scale [8] when this instrument is used to study the associations and relations between motivation and outcome variables (like performance) or influencing variables (like age and gender), and not in high stakes situations (like medical school admissions). This concept of validity as validity of the scores of the instrument rather than the instrument itself, is in line with the definition specified by Downing, American Educational Research Association (AERA) and the American Psychological Association (APA) [11, 12] Values of Cronbach’s alpha of the subscales and the overall instrument were 0.70, 0.67, 0.55 and 0.79 respectively. The strength of motivation for medical school positively correlated with another measure of academic motivation (Academic Motivation Scale) and study stress (measured by Maslach Burnout Inventory-Student Survey). The SMMS-R has been utilized to examine demographic differences in students’ motivation for medical school between different age, gender and educational background groups [5–7].

Although the Dutch version of the SMMS-R has been validated in medical students, its measurement invariance has not yet been examined. Moreover, studies of motivation for medical school across countries can be legitimized only after the equivalence of other language versions including the Dutch version is identified.

After an instrument is administered, comparison is a common approach in order to find out if there is a significant difference between groups (e.g., gender, ethnicity, and cultures). On the one hand, this difference might be a true existence, on the other hand, it can be caused by the instrument itself, that is, different groups might perceive the same instrument differently, in this case, a single item of an instrument in fact appears to have different meanings to different groups. Measurement invariance (MI) is established when items of an instrument have equivalent meanings across groups being compared [13], i.e., when a questionnaire measures identical constructs within the same structure across different groups it is called measurement invariant [14]. With demonstration of MI for a questionnaire, one can conclude that the participants across groups interpret the individual questions as well as the underlying latent factor in the same way [14]. MI is a pre-requisite for valid comparison between groups in order to ensure equity and fairness in selection based on the measure [15].

MI comprises configural, metric and scalar invariance.

*Configural invariance* is the first step to be tested, it can be established when the measurement scale has a similar factor structure across groups, i.e., same factors include same questions [16]. It can be tested by imposing the same structure across groups and allowing all model estimated parameters to differ.

*Metric invariance* Once the configural invariance is satisfied, one can further test its metric invariance, the determination as to whether different groups have the same factor loadings for the same item.

*Scalar invariance* is usually the last step for establishing measurement invariance in practice [17], in addition to the requirements aforementioned, it examines whether different groups have the same item intercepts. To do so, researchers can set intercepts and factor loadings for the same item to be equal across groups.

Since the full measurement invariance is too strict to hold in practice, partial measurement invariance was introduced [18]. Instead of requiring all estimated parameters to be invariant, partial MI relaxes some of these parameters to differ across groups. Van den Berg and Lance (2000) suggested that partial MI should be examined only on a small proportion of indicators, and there should have strong theoretical or practical justifications of doing so [19].

Considering that the measurement invariance of the SMMS-R has not been examined yet, the aims of this study were to verify the factorial structure of the SMMS-R, and to test its group invariance in the Chinese context.

## Methods

### Study design and setting

This study was a cross-sectional study and data was collected through paper-based questionnaires distributed at Shantou University Medical College, China. Shantou University Medical College (SUMC) is an important part of Shantou University, a public university jointly funded by the government and Li Ka Shing Foundation. SUMC offers a 5 + 3 Joint Bachelor-Master program and a series of master’s, doctoral and post-doctoral programs in medicine. It has five affiliated hospitals with 4419 patient beds and five associated partner hospitals with 5499 patient beds.

### Sample

Study participants were 989 students from SUMC. Participants’ ages ranged from 18 to 22 years (mean age = 20.05 years, standard deviation = 1.86).

Given the interest in comparisons of motivations for medical schools between samples in China (e.g., students from schools in different regions or classified at different levels), we initially wanted to test the measurement invariance across two samples. Since another sample was difficult to organize, we adopted an accepted alternative approach called sample splitting which has been used before in other studies [20, 21]. Following the same manner, we randomly divided the whole sample into two halves.

The sample was then divided into two halves by random sampling stratified by college year (464 in Group 1, and 466 in Group 2) for the purpose of the investigation into measurement invariance.

### Instruments

The SMMS-R was employed to measure participants’ motivation for medical college, as aforementioned in the introduction section, initial evidence of reliability [6–8] and validity of the scores using the SMMS-R was obtained in previous studies [8]. Exploratory factor analysis (EFA) suggested a 3-factor structure, and the reported Cronbach’s alphas for the full instrument and three subscales were acceptable.

Three translation stages were conducted to ensure language equivalence [22]. First, two team members translated the SMMS-R from English into Chinese independently. Agreement was reached by discussions on translation results and consensus. The Chinese version was then translated back into English by a team member fluent in English. Finally, the principal investigator compared the back-translated English version with the Original SMMS-R, and semantic difference in translations were discussed and resolved with team members.

### Data collection

The Chinese version of the SMMS-R was administered to 989 college students. 59 cases were discarded due to incomplete data (e.g., missing values, incompleteness, errors, etc.), 930 cases were included in the final analysis.

### Statistical analyses

In this study, we conducted the single and multiple group confirmatory factor analyses to further examine the factorial validity and measurement invariance of the scores on three SMMS-R subscales, and the full scale. Proposed structural models are tested against the null model (or the baseline model in which all of the variables are uncorrelated) to see whether they are statistically superior. Model fit indices include: the Chi-square statistic and associated probability, Comparative Fit Index (CFI), Tucker-Lewis Index (TLI), Sstandardized Root Mean Square Residual (SRMR), and Root Mean Square Error of Approximation (RMSEA). CFI ≥ 0.96, TLI ≥ 0.95, SRMR ≤ 0.08, and RMSEA ≤ 0.06 were considered indicative of good model fit [14, 23], and were considered acceptable when CFI ≥ 0.90, TLI ≥ 0.90, SRMR ≤ 0.10, and RMSEA ≤ 0.08 [14, 19].

Given the fact that Chi-square test is strongly influenced by sample size [16, 24], we adopted to observe the change of CFI between nested models in order to assess measurement invariance. Change in CFI equal or less than 0.01 is considered acceptable [16].

Internal consistency was measured by Cronbach’s alpha along with McDonald’s ω and glb. McDonald’s ω and glb are advocated as better alternative estimators with obvious advantages compared to alpha [25, 26].

Statistical analyses were conducted using SPSS version 20.0 and Mplus version 7 for windows. FACTOR software was used to compute ω and glb [27].

### Ethical approval

Prior to the implementation, this study was reviewed and approved by the institutional Human Research Ethics Committee (Ref No. SUMC-2016-31). Details and objectives were explained to participants and consent forms were obtained before data collection. Participation was voluntary and the participants were explained that non-participation did not carry any consequences for them. The participants were also assured that the analysis would be conducted after anonymizing the data and that the results would be reported only at a group level.

## Results

### Descriptive statistics for participants information and motivation scores

The sample used in the present study is representative, as the gender ratio among the participants was 50.2% males (492/989) and 49.8% females (438/989), which is similar with the actual ratio (the difference was less than 5%) in the whole population.

Mean ages for the two groups were 20.13 years (SD = 1.43) and 19.97 years (SD = 2.20). Chi-square test for categorical variables (e.g., gender) and t-test for continuous variables (e.g., age) were implemented, and there were no demographic differences between these two samples. Please see Table 1.

**Table 1:** Sample characteristics

Variable	Gender, n (%)		Age, yrs.	
	Male	Female	Mean	SD
Group 1 (*n* = 464)	248	216	20.13	1.43
Group 2 (*n* = 466)	244	222	19.97	2.20
Total (*n* = 930)	492	438	20.05	1.86
Test statistic for sample difference	χ^2^ (1, *n* = 930) = 0.74, *p* = 0.11		*t *(928) = 1.24, *p* = 0.216	

The means for each subscale, created by averaging the items, and standard deviations for each group are reported in Table 2. Distributions of total and subscale scores are broadly normal, please see distribution histograms in the Additional file 1. A correlation matrix and a covariance matrix reflecting relationships between SMMS-R subscale scores for the whole group were also computed, please see Additional file 2.

**Table 2:** Descriptive Statistics for the SMMS Total and Subscale Scores

Group	Willingness to sacrifice subscale		Readiness to start subscale		Persistence subscale		Total score	
	Mean	SD	Mean	SD	Mean	SD	Mean	SD
Group 1 (*n* = 464)	3.41	0.61	3.01	0.80	3.30	0.66	3.24	0.52
Group 2 (*n* = 466)	3.47	0.64	3.04	0.78	3.38	0.64	3.30	0.51
Male (*n* = 492)	3.36	0.64	3.00	0.78	3.31	0.68	3.23	0.52
Female (*n* = 438)	3.53	0.60	3.05	0.79	3.37	0.62	3.32	0.50
Total (*n* = 930)	3.44	0.63	3.03	0.79	3.34	0.65	3.27	0.51

### Reliability

Internal consistency for all three subscales and the whole instrument was acceptable. Cronbach’s alpha values were 0.66, 0.79 and 0.61 for willingness to sacrifice, readiness to start, and persistence subscales respectively and 0.79 for the full SMMS-R. The range of these estimates is consistent with the values reported in the original study [8]. McDonald’s omegas were 0.68 for the willingness to sacrifice subscale, 0.79 for the readiness to start subscale, and 0.62 for the persistence subscale. Finally, glbs were 0.70, 0.80, and 0.66 for three subscales respectively. The global SMMS-R glb was 0.86. Results are presented in Table 3.

**Table 3:** Fit statistics for scales from the confirmatory factor analyses

Scale	Number of items	χ^2^	*df*	*P*	CFI	TLI	SRMR	RMSEA	RMSEA 90% CI	α	ω	glb
Full scale with unidimensional solution	15	1990.97	90	0.0000	0.664	0.608	0.09	0.11	[0.10 0.12]			
Full scale initial	15	417.67	87	0.0000	0.889	0.866	0.06	0.06	[0.06 0.07]			
Full scale revised	15	332.59	85	0.0000	0.917	0.897	0.05	0.05	[0.05 0.06]	0.79	0.77	0.86
F1	5	7.09	5	0.2141	0.997	0.993	0.01	0.02	[0.00 0.05]	0.66	0.68	0.70
F2	5	23.32	5	0.0003	0.984	0.969	0.02	0.06	[0.04 0.09]	0.79	0.79	0.80
F3	5	15.91	4	0.0031	0.974	0.935	0.02	0.06	[0.03 0.09]	0.61	0.62	0.66

*F1* willingness to sacrifice subscale, *F2* readiness to start subscale, *F3* persistence subscale, *CFI* comparative fit index, *TLI* Tucker-Lewis Index, *CI* confidence interval, *SRMR* standardized root-mean-square residual *RMSEA* root-mean-square error of approximation *α* Cronbach’s alpha; *ω* McDonald’s omega; *glb* greatest lower bound. Full scale initial means a prior three-factor model was fitted to the data; Full scale revised means initial model was revised by specifying the residual correlations between e9 and e4, and between e14 and e13

### Model fit

A single dimension model was fitted to the data, i.e., all 15 SMMS-R items were specified to load on a single factor. This is not a good solution as model fit indices are all unacceptable. We presented evidence to show that a 3-factor solution for the SMMS-R rather than an unidimensional factor structure is statistically superior (χ2(90) = 1990.97, *p* < 0.01, CFI = 0.664, TLI = 0.608, SRMR = 0.09, and RMSEA = 0.11, Table 3, second row).

Confirmatory factor analyses on the full SMMS-R were then performed with the entire sample to verify its 3-factor structure, and initial results showed that the model was not a good fit to the data. After a closer inspection of the modification indices and specifying the residual correlations between e9 and e4, and between e14 and e13 (residuals of factor indicators are un-correlated is a default setting in Mplus), acceptable model data fit was achieved (Table 2). We have a theoretical justification for doing so because e4 and e9 are items related to time-consuming reasons, and e13 and e14 are related because they are concerning livelihood, they are theoretically correlated. Overall fit indices supported this 3-factor structure of the SMMS-R: χ2(85) = 332.59, *p* < 0.01, CFI = 0.917, TLI = 0.897, SRMR = 0.05, and RMSEA = 0.05, indicating that the model is an adequate representation of the observed data. A graphic display of this final structural model with standardized parameter estimators are presented in the Additional file 3.

Single group confirmatory factor analysis was then employed on each individual subscale, and fit indices throughout each subscales showed acceptable model data fit (Table 2). Chi-square values for the readiness to start subscale (χ2(*df*) = 23.32(5), *p* = 0.000) and the persistence subscale (χ2(*df*) = 15.91(4), *p* = 0.003) were significant, and according to previous studies [28, 29], the Chi-square value is reasonable when a model has 75–200 observations, and is almost always statistically significant when a model has over 400 observations. Since we have 930 participants and 400 cases in each group, we decided to adopt CFI, TLI, SRMR, and RMSEA as model fit indices.

### Measurement invariance of SMMS-R

Configural invariance was first examined by specifying the same structure across different groups while allowing all other parameters to differ. The fit indices for this model were acceptable across gender groups (CFI = 0.91; TLI = 0.90; SRMR = 0.06; RMSEA = 0.06), and across sample groups (CFI = 0.92; TLI = 0.90; SRMR = 0.06; RMSEA = 0.06), suggesting that participants from these different groups conceptualize the three-dimensional structure of motivation for medical schools in the same way. Thus, configural invariance as a baseline model for each subscale was established.

Metric invariance was then tested by requiring the same factor structure and equal factor loadings across groups while all other parameters were allowed to differ. The fit indices were all good, and the changes in CFI across different were all less than 0.01, indicating the null hypothesis of metric invariance should be accepted, meaning that metric invariance for the whole instrument was demonstrated.

Scalar invariance was the final form of measurement invariance to be tested in our study, by constraining equal item intercepts, factor loadings across different groups while allowing other parameters to differ. As Table 4 shows, fit values were all located within acceptable range, changes in CFI were all 0.00, indicating participants who obtain score similarly on a latent construct would also score in the same way irrespective of the group they belong to, which means group comparison of means make sense.

**Table 4:** Measurement invariance for the whole SMMS-R

Model	χ^2^	*df*	*p*	△χ^2^(*p*)	CFI	△CFI	TLI	SRMR	RMSEA	RMSEA 90% CI
Across gender groups										
Step 1: Configural invariance	441.58	170	0.0000		0.91		0.90	0.06	0.06	[0.05, 0.07]
Step 2: Metric invariance	452.09	182	0.0000	−10.51 (0.0000)	0.91	0.00	0.90	0.06	0.06	[0.05, 0.06]
Step 3: Scalar invariance	479.74	194	0.0000	−27.65 (0.0000)	0.90	0.01	0.90	0.06	0.06	[0.05, 0.06]
Across sample groups										
Step 1: Configural invariance	424.90	170	0.0000		0.92		0.90	0.06	0.06	[0.05, 0.06]
Step 2: Metric invariance	443.56	182	0.0000	−19.65 (0.0000)	0.91	0.01	0.90	0.06	0.06	[0.05, 0.06]
Step 3: Scalar invariance	455.69	194	0.0000	−12.13 (0.0000)	0.91	0.00	0.91	0.06	0.05	[0.05, 0.06]

*CFI* comparative fit index; *TLI* Tucker-Lewis Index; *CI* confidence interval; *SRMR* standardized root-mean-square residual; *RMSEA* root-mean-square error of approximation

### Measurement invariance of subscales of the SMMS-R

Since the SMMS-R are often used to compare students’ motivation at the subscale level, we further test its measurement invariance for each subscale separately. Following the same procedure, we further examined measurement invariance for each subscale of the SMMS-R across gender groups (Table 5) and two sample groups (Table 6). Among the results, fit values were all located within acceptable range, changes in CFI were less than 0.01 except a change of 0.02 was observed for the Persistence subscale when measuring scalar invariance (Table 5). According to modification indices, intercepts for item 4 and item 8 were allowed to differ. By doing so, a change in CFI was less than 0.01, supporting partial scalar invariance.

**Table 5:** Measurement invariance for subscales across gender

Scale	χ^2^	*df*	*p*	△χ^2^ (*p*)	CFI	△CFI	TLI	SRMR	RMSEA	RMSEA 90% CI
F1										
Step 1: Configural invariance	12.41	10	0.2584		1.00		0.99	0.02	0.02	[0.00, 0.06]
Step 2: Metric invariance	15.80	14	0.3256	−3.39 (−0.0672)	1.00	0.00	1.00	0.03	0.02	[0.00, 0.05]
Step 3: Scalar invariance	20.45	18	3081	−4.65 (−0.175)	1.00	0.00	1.00	0.03	0.02	[0.00, 0.05]
F2										
Step 1: Configural invariance	25.21	10	0.0050		0.99		0.97	0.02	0.06	[0.03, 0.08]
Step 2: Metric invariance	31.06	14	0.0054	−5.85 (−0.0004)	0.99	0.00	0.98	0.04	0.05	[0.03, 0.08]
Step 3: Scalar invariance	44.42	18	0.0005	−13.36 (0.0050)	0.98	0.01	0.98	0.04	0.06	[0.04, 0.08]
F3										
Step 1: Configural invariance	21.07	8	0.0070		0.97		0.93	0.03	0.06	[0.03, 0.09]
Step 2: Metric invariance	27.32	12	0.0069	−6.25 (0.0001)	0.97	0.00	0.94	0.04	0.05	[0.03, 0.08]
Step 3: Scalar invariance	39.17	16	0.0010	−11.85 (0.0059)	0.95	0.02	0.94	0.04	0.06	[0.03, 0.08]
Step 4: intercepts freed to vary	31.08	14	0.0054	−3.76 (0.0015)	0.96	0.01	0.95	0.04	0.06	[0.03, 0.08]

*F1* willingness to sacrifice subscale, *F2* readiness to start subscale *F3* persistence subscale *CFI* comparative fit index, *TLI* Tucker-Lewis Index, *CI* confidence interval, *SRMR* standardized root-mean-square residual, *RMSEA* root-mean-square error of approximation

**Table 6:** Measurement invariance for scales across two independent samples

Scale	χ^2^	*df*	*p*	△χ^2^ (*p*)	CFI	△CFI	TLI	SRMR	RMSEA	RMSEA 90% CI
F1										
Step 1: Configural invariance	8.86	10	0.5446		1.00		1.00	0.02	0.00	[0.00, 0.05]
Step 2: Metric invariance	13.76	14	0.4676	−4.90 (0.0770)	1.00	0.00	1.00	0.03	0.00	[0.00, 0.04]
Step 3: Scalar invariance	16.78	18	0.5381	−3.02 (−0.0705)	1.00	0.00	1.00	0.03	0.00	[0.00, 0.04]
F2										
Step 1: Configural invariance	25.37	10	0.0047		0.99		0.97	0.02	0.06	[0.03, 0.09]
Step 2: Metric invariance	29.20	14	0.0098	−3.83 (−0.0051)	0.99	0.00	0.98	0.03	0.05	[0.02, 0.07]
Step 3: Scalar invariance	33.97	18	0.0127	−4.77 (−0.0029)	0.99	0.00	0.99	0.04	0.04	[0.02, 0.07]
F3										
Step 1: Configural invariance	21.91	8	0.0051		0.97		0.93	0.03	0.06	[0.03, 0.09]
Step 2: Metric invariance	27.44	12	0.0067	−5.53 (−0.0016)	0.97	0.00	0.95	0.04	0.05	[0.03, 0.08]
Step 3: Scalar invariance	30.28	16	0.0166	−2.84 (−0.0099)	0.97	0.00	0.96	0.04	0.04	[0.02, 0.07]

*F1* willingness to sacrifice subscale, *F2* readiness to start subscale, *F3* persistence subscale *CFI* comparative fit index, *TLI* Tucker-Lewis Index, *CI* confidence interval, *SRMR* standardized root-mean-square residual, *RMSEA* root-mean-square error of approximation

In summary, adequate model fit was demonstrated by multi-group CFA analyses.

## Discussion

This study reports the measurement invariance of the SMMS-R across genders and two independent samples. To our knowledge, this has not been done before.

Although an original study suggested a unidimensional solution [9], a 3-factor structure was reported to be more theoretically and empirically appropriate in a subsequent study [8], in this study we provide further evidence to support this 3-factor structure as the fit indices of CFA analyses for each subscale of the Chinese version of the SMMS-R were acceptable.

Full configural and metric invariance of the SMMS-R across all groups were obtained for all the subscales. Scalar invariance was identified for the “willingness to sacrifice” and “readiness to start” subscales across gender groups, after allowing intercepts of item 4 and item 8 to vary, partial scalar invariance was established for the “persistence” subscale. Because all models (configural, metric, and scalar invariance) showed adequate fit estimates, it is appropriate to conclude that the SMMS-R is invariant across groups, and when group differences are identified utilizing the SMMS-R, they are more likely to be real differences rather than the differences caused by different interpretations of the instrument by the participants.

Our findings about reliability for each subscale ranged from 0.61 to 0.79, indicating acceptable internal consistencies as they were above the lower limit of 0.6 [30]. In line with previous findings [5–8], the persistence subscale has the lowest value of Cronbach’s alpha, and the full instrument’s internal consistency (Cronbach’s alpha is 0.79) is identical with findings in past studies [6–8]. In addition, the reliability for the persistence subscale in this study is slightly greater than the previous findings, given that Cronbach’s alpha is strongly influenced by the length of the test [30], we recommend to include more items in this subscale in order to improve it’s reliability in future studies.

### Implications for future research and practice

The SMMS-R can be further used to study the association of motivation, learning, performance and well-being of medical students in non-Western contexts. Longitudinal studies investigating of the strength of motivation over the period of the medical course would provide a deeper understanding of the concept of strength of motivation. As concluded by Leibach and Stern [10], this would enable administrators and faculty members to help students who may need support and interventions to improve their strength of motivation especially when they face adverse circumstances during their study.

### Limitations

Cross-sectional data was used and measurement invariance was demonstrated between groups, in other words, the SMMS-R can be used to make comparisons across groups. However, this study did not test its longitudinal measurement invariance. We recommend further research using the SMMS-R to observe a change over time within a group. Finally, majority of our sample population are Han Chinese. Participants from Western areas such as Xinjiang and Tibet where the most ethic minority Chinese live were not included in this study. Further tests on measurement invariance across races and other contexts are recommended.

## Conclusions

In sum, the 15-item SMMS-R allows for measurement of three dimensions of motivation for medical education, this three-factor structure also has an acceptable model-data fit within a Chinese context. Good evidence of measurement invariance were demonstrated by multi-group confirmatory factor analyses across gender and independent sample groups. Therefore it can be used for group and cross-cultural comparisons.

## Additional files

Additional file 1: Distributions of the scale scores. (DOCX 55 kb)

Additional file 2: Correlation matrix and covariance matrix. (DOCX 12 kb)

Additional file 3: Final factor structure of the SMMS-R. (PNG 47 kb)
