# Supplementary figures and images for: Measurement invariance of the strength of motivation for medical school: a multi-group confirmatory factor analysis
Source: BMC Med Educ. 2017 Jul 11;17:116. doi: 10.1186/s12909-017-0958-4 (PMC5505130; doi:10.1186/s12909-017-0958-4)

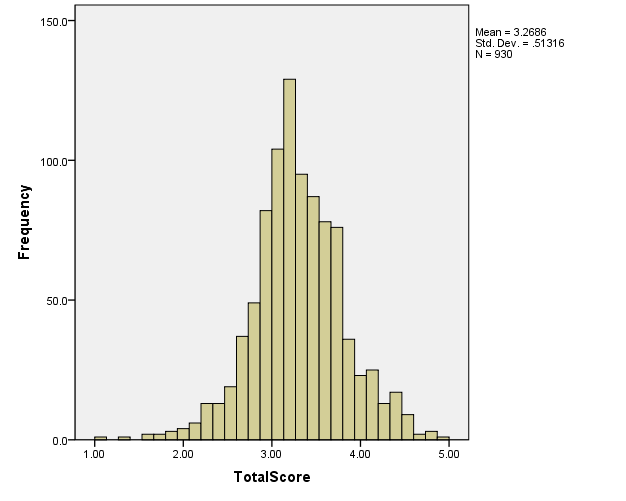


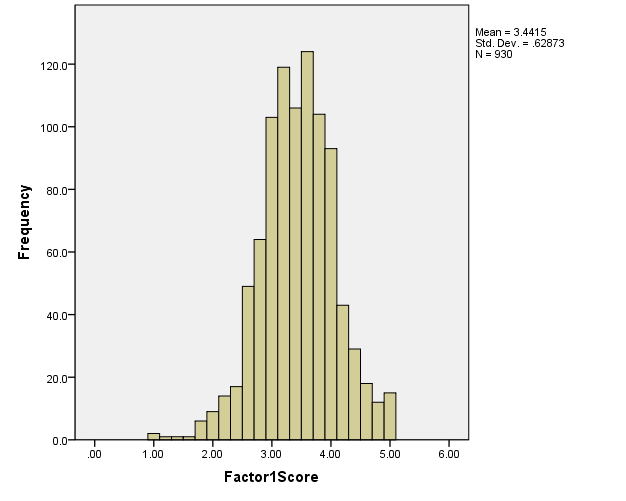


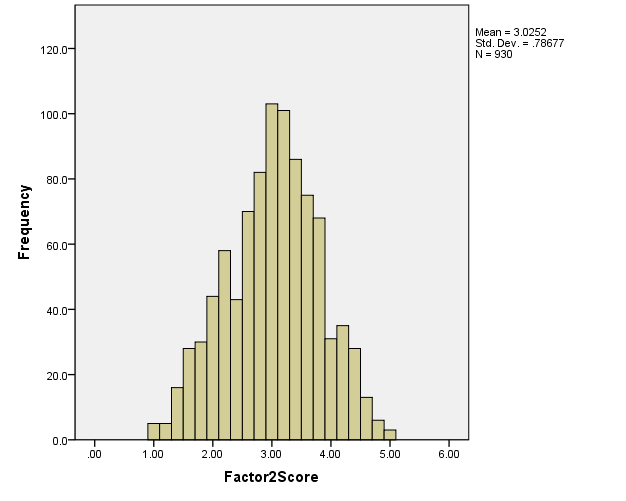


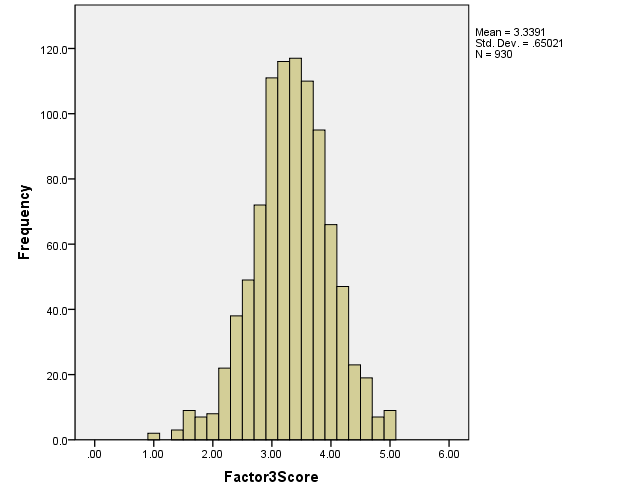

Supplement: Supplementary file 1 — Distributions of the scale scores. (DOCX 55 kb) [file 12909_2017_958_MOESM1_ESM.docx]

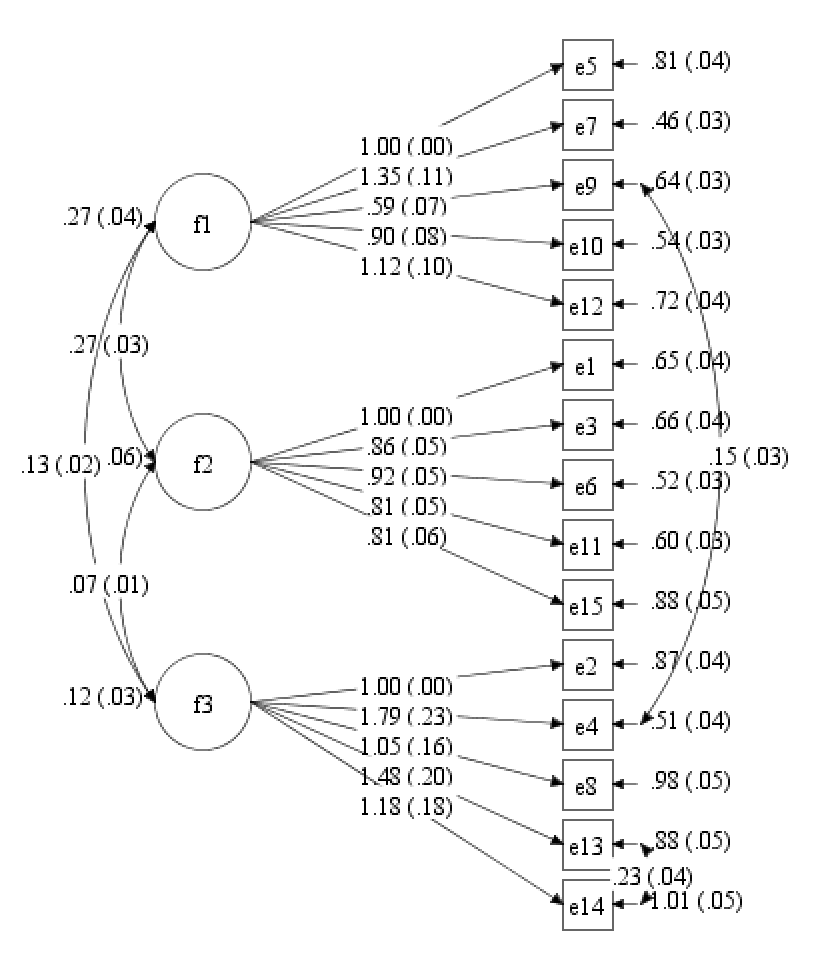

Supplement: Supplementary file 3 — Final factor structure of the SMMS-R. (PNG 47 kb) [file 12909_2017_958_MOESM3_ESM.png]
