# Supplementary material for: Measurement invariance of the strength of motivation for medical school: a multi-group confirmatory factor analysis
Source: BMC Med Educ. 2017 Jul 11;17:116. doi: 10.1186/s12909-017-0958-4 (PMC5505130; doi:10.1186/s12909-017-0958-4)
Supplement: Supplementary file 2 — Correlation matrix and covariance matrix. (DOCX 12 kb) [file 12909_2017_958_MOESM2_ESM.docx]

Correlation Matrix of the SMMS subscale scores

|  | Subscale 1 | Subscale 2 | Subscale 3 |
| --- | --- | --- | --- |
| Subscale 1 | 1.00 |  |  |
| Subscale 2 | 0.45 | 1.00 |  |
| Subscale 3 | 0.45 | 0.12 | 1.00 |

Covariance Matrix of the SMMS subscale scores

|  | Subscale 1 | Subscale 2 | Subscale 3 |
| --- | --- | --- | --- |
| Subscale 1 | 0.40 |  |  |
| Subscale 2 | 0.22 | 0.62 |  |
| Subscale 3 | 0.18 | 0.06 | 0.42 |
